# Supplementary material for: OpenMM 7: Rapid development of high performance algorithms for molecular dynamics
Source: PLoS Comput Biol. 2017 Jul 26;13(7):e1005659. doi: 10.1371/journal.pcbi.1005659 (PMC5549999; doi:10.1371/journal.pcbi.1005659)
Supplement: S1 Source Code — (ZIP) [file pcbi.1005659.s002.zip › OpenMM-7.0.1-Source/docs/api-c++/generated/OpenMM.VariableVerletIntegrator.html]

VariableVerletIntegrator — OpenMM @OPENMM\_MAJOR\_VERSION@.@OPENMM\_MINOR\_VERSION@ documentation


# VariableVerletIntegrator¶

*class* `OpenMM::``VariableVerletIntegrator`¶
:   This is an error contolled, variable time step `Integrator` that simulates a `System` using the leap-frog Verlet algorithm. It compares the result of the Verlet integrator to that of an explicit Euler integrator, takes the difference between the two as a measure of the integration error in each time step, and continuously adjusts the step size to keep the error below a specified tolerance. This both improves the stability of the integrator and allows it to take larger steps on average, while still maintaining comparable accuracy to a fixed step size integrator.

    It is best not to think of the error tolerance as having any absolute meaning. It is just an adjustable parameter that affects the step size and integration accuracy. You should try different values to find the largest one that produces a trajectory sufficiently accurate for your purposes. 0.001 is often a good starting point.

    Unlike a fixed step size Verlet integrator, variable step size Verlet is not symplectic. This means that at a given accuracy level, energy is not as precisely conserved over long time periods. This makes it most appropriate for constant temperate simulations. In constant energy simulations where precise energy conservation over long time periods is important, a fixed step size Verlet integrator may be more appropriate.

    Methods

    |  |  |
    | --- | --- |
    | `VariableVerletIntegrator` | Create a `VariableVerletIntegrator`. |
    | `getErrorTolerance` | Get the error tolerance. |
    | `setErrorTolerance` | Set the error tolerance. |
    | `step` | Advance a simulation through time by taking a series of time steps. |
    | `stepTo` | Advance a simulation through time by taking a series of steps until a specified time is reached. |

    `VariableVerletIntegrator`(double *errorTol*)¶
    :   Create a `VariableVerletIntegrator`.

        |  |  |
        | --- | --- |
        | Parameters: |  |

        - **errorTol** – the error tolerance

    double `getErrorTolerance`() *const*¶
    :   Get the error tolerance.

    void `setErrorTolerance`(double *tol*)¶
    :   Set the error tolerance.

    void `step`(int *steps*)¶
    :   Advance a simulation through time by taking a series of time steps.

        |  |  |
        | --- | --- |
        | Parameters: |  |

        - **steps** – the number of time steps to take

    void `stepTo`(double *time*)¶
    :   Advance a simulation through time by taking a series of steps until a specified time is reached. When this method returns, the simulation time will exactly equal the time which was specified. If you call this method and specify a time that is earlier than the current time, it will return without doing anything.

        |  |  |
        | --- | --- |
        | Parameters: |  |

        - **time** – the time to which the simulation should be advanced

High performance molecular simulation on GPUs


### Navigation

- Core Objects
- Forces
- Integrators
- Extras

©2015, Stanford University and the Authors.
|
Powered by Sphinx 1.3.1
& Alabaster 0.7.7
|
Page source
